# Factors associated with overall and high-risk return visits to the emergency department: a vital sign trajectory approach

**DOI:** 10.1186/s12873-025-01211-1

**Published:** 2025-04-12

**Authors:** Hsiao-Chia Wang, Cheng-Chung Fang, Chien-Hua Huang, Jun-Wan Gao, Jiann-Hwa Chen, Chu-Lin Tsai

**Affiliations:** 1 Department of Emergency Medicine, Cathay General Hospital, No. 280, Sec. 4, Renai Rd., Taipei City, 106 Taiwan; 2 School of Medicine, Fu Jen Catholic University, New Taipei City, Taiwan; 3 Department of Emergency Medicine, National Taiwan University Hospital, 7 Zhongshan S. Rd, Taipei City, 100 Taiwan; 4 Department of Emergency Medicine, College of Medicine, National Taiwan University, Taipei City, Taiwan

**Keywords:** Emergency department, Revisit, Group-based trajectory modeling

## Abstract

**Background:**

For patients and emergency department (ED) physicians, return visits to the ED represent a potentially detrimental issue. In this study, our goal was to examine factors associated with overall and high-risk ED revisits. Specifically, as vital signs during the ED stay may provide important clues for subsequent revisits, we also examined the association between vital sign trajectories and post-ED revisits.

**Methods:**

This retrospective cohort study utilized electronic clinical warehouse data from a tertiary medical center. We retrieved data from 454,330 ED visits over four years. The data included patient demographics, triage data, and repeated vital sign measurements. Group-based trajectory modeling was used to identify vital sign trajectories. A high-risk return ED visit was defined as a revisit within 72 h of the index visit with intensive care unit admission, receiving emergency surgery, or with in-hospital cardiac arrest. Multivariable logistic regression analysis was performed to evaluate the associations between vital sign trajectories and revisits.

**Results:**

A total of 39,138 potential index ED visits were analyzed. Of these, 3,201 resulted in revisits, accounting for an 8.2% overall revisit rate and a 0.2% high-risk revisit rate. A high but resolving body temperature trajectory was associated with overall revisits (adjusted odds ratio [aOR], 1.32; 95% confidence interval [95% CI], 1.13–1.53). By contrast, high-risk revisits were associated with a low/fluctuating oxygen saturation trajectory (aOR, 2.40; 95% CI, 1.15–4.99). Older age (aOR, 1.27 per 10-year increase; 95% CI, 1.11–1.46) and having a chronic major disease (aOR, 2.30; 95% CI, 1.38–3.84) were also associated with high-risk revisits.

**Conclusions:**

In addition to older age and having a chronic major disease, a low and fluctuating oxygen saturation trajectory during the index ED stay may signal subsequent high-risk revisits. Thus, discharge decisions should be carefully re-evaluated in these high-risk populations.

**Supplementary Information:**

The online version contains supplementary material available at 10.1186/s12873-025-01211-1.

## Introduction

After receiving initial care in the emergency department (ED), most patients are discharged to continue their recovery at home. However, a noteworthy percentage of patients, ranging from 5 to 10%, return to the ED within three days [1–3]. These return visits not only pose a burden on healthcare resources but also lead to significant costs. A previous study revealed that the cumulative cost of return ED visits surpassed the total expenses incurred during initial visits [1]. Recognizing the clinical and financial implications, the rate of return to the ED has become a commonly used quality measure, assuming that it can reflect the standard of care provided during the initial visit.

However, recent data challenge this assumption by showing that only a modest percentage, approximately 5–10%, of return visits are directly associated with prior medical care [4–8]. Patient-related factors, including uncertainty about disease progression, treatment preferences, and natural disease evolution, also contribute to ED return visits [6]. Consequently, recent studies have proposed alternative quality metrics, focusing on patient outcomes after post-return ED visits, such as high-risk return visits (e.g., return intensive care unit [ICU] admissions). Previous studies have found that patients who were hospitalized after a return visit to the ED did not necessarily have worse outcomes compared to those who were directly admitted [9, 10], suggesting that return admissions may not reflect care issues in prior ED visits. By contrast, a study investigating ICU admissions post-ED revisit uncovered a noteworthy yield (14%) in screening for potential medical deficiencies [11]. Notably, our previous study utilized a case-crossover design to examine within-person changes related to these high-risk return ED visits [12]. As vital signs during the ED stay may provide important clues for subsequent revisits, we are interested in examining the relationship between vital sign trajectories and post-ED high-risk revisits. To our knowledge, no studies have employed trajectory modeling to study these high-risk return ED visits. Understanding these vital sign changes may help with the early recognition and prevention of these catastrophic events.

In this study, we aimed to investigate the factors associated with overall return visits and high-risk revisits. Specifically, we used trajectory modeling to examine the associations between vital sign trajectories during the index ED visits and post-ED overall and high-risk revisits.

## Methods

### Study design and setting

We conducted a retrospective cohort study using electronic health record (EHR) data from the Cathay General Hospital (CGH) System. The system included a main hospital along with two branch hospitals. The CGH main hospital is a tertiary medical center with 800 beds and approximately 60,000 ED visits per year, with two branch regional hospitals collectively serving approximately 100,000 ED visits. This database serves as a central clinical data warehouse for all electronic health records in the healthcare system, encompassing inpatient, outpatient, and ED records. The electronic database contains various information, including demographics, diagnosis, treatment, imaging, laboratory, prescription, nursing, billing, and administrative data. The database is maintained and updated by dedicated research personnel.

For the current study, we retrieved four years of de-identified data between January 1, 2016, and December 31, 2019. All CGH data are de-identified but contain a unique, encrypted personal identifier, enabling researchers to link visit records. This study was approved by the CGH Institutional Review Board, which waived the requirement for patient informed consent.

### Study population

**Fig. 1:**
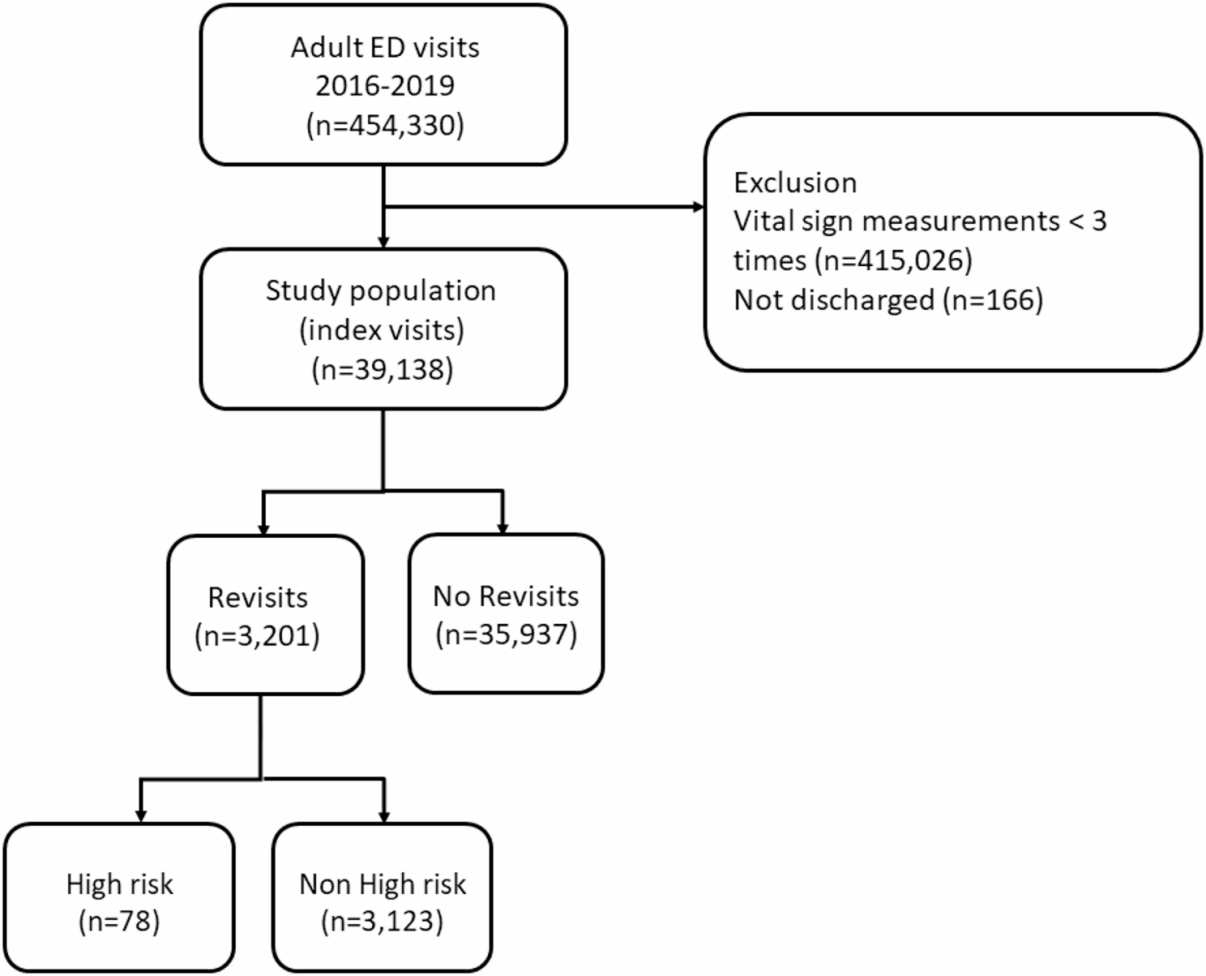
Flow diagram of the patient selection process. Abbreviations: ED, emergency department

Data from 454,330 adult ED visits (aged 18 and older) were electronically extracted over the 4-year period. For the current analysis, due to our interest in the predictive role of vital sign trajectories during the ED stay, we excluded individuals with fewer than three vital sign measurements. At least three measurements ensured the stability of the trajectory analysis. We further excluded those who were hospitalized because they would not serve as the “index” ED visits. The index ED visits were the initial “treat-and-release” ED visits. A return ED visit was defined as an ED revisit within 72 h of the index visit. Two types of return ED visits were examined in the current analysis: (1) the overall revisit population and (2) the high-risk revisit population, which was defined as a subgroup of revisit patients who developed severe adverse outcomes, including intensive care unit (ICU) admissions, emergency surgery within 24 h of ED revisit, or in-hospital cardiac arrest (IHCA) receiving cardiopulmonary resuscitation during the return visit. For multiple revisits within 72 h, we only selected the first revisit. The subject selection process is illustrated in Fig. 1.

### Variables

Patient demographics and clinical information with timestamps in the ED were extracted, including chief complaints on ED presentation, mode of arrival, transfer status, and serial vital sign measurements (systolic blood pressure [SBP], diastolic blood pressure [DBP], heart rate [HR], body temperature [BT], respiratory rate [RR], and oxygen saturation [SpO_2_]). The vital signs were measured from hour 0 (at ED triage) to hour 6, as most of the discharged patients stayed in the ED for less than 6 h. The vital sign data were split into 1-hour blocks. If multiple measurements were recorded in the 1-hour block, the average value was computed and used.

Emergency department shifts were classified as day (07:00–14:59), evening (15:00–22:59), and night (23:00–06:59) shifts. We also extracted the five-level computerized Taiwan Triage and Acuity Scale (TTAS) data, which is decision-path software that begins with a total of 179 structured chief complaints. Based on the TTAS computerized algorithms, patients are prioritized in the following order of acuity: level 1, resuscitation; level 2, emergent; level 3, urgent; level 4, less urgent; and level 5, non-urgent. The TTAS was adapted from the Canadian Triage and Acuity Scale and has been validated against hospitalization, ED length of stay, and resource use [13]. Major disease diagnoses, such as cancer, end-stage renal disease, and ventilator dependence, were defined by the National Health Insurance in Taiwan (Online Supplementary Table S1). The age and sex of the treating physicians were also extracted.

The data extraction was performed by hospital information technology engineers who were blinded to the study hypothesis. The data underwent electronic cleaning, and invalid data were set to missing values after periodic discussions at investigator meetings. The data cleaning process involved mostly vital sign variables, with less than 1% being out of range. For example, we defined the respiratory rate as ranging between 0 and 50 per minute. Invalid vital sign data were set to missing values.

### Outcome measures

The primary outcome measure was the high-risk revisit rate, calculated by dividing the number of high-risk revisits by the total number of discharged index ED visits. The coprimary outcome measure was the overall ED revisit rate, which was calculated as the number of all revisits divided by the total number of discharged index ED visits.

### Statistical analysis

Summary statistics are displayed as proportions (with 95% confidence intervals [CIs]), means (with standard deviations [SDs]), or medians (with interquartile ranges [IQRs]). Patient characteristics were compared between the revisit status groups. Univariate associations were examined using Student’s t-tests, Mann-Whitney tests, and chi-square tests, as appropriate.

Group-based trajectory modeling (GBTM) was conducted to identify trajectory groups within each of the six vital sign categories. GBTM is an explanatory modeling approach to identifying underlying groups of individuals with similar trajectories for a particular variable of interest [14]. This technique employs finite mixture modeling to identify clusters of longitudinal data [15]. We fit models of two to six trajectory groups, including constant, linear, quadratic, or cubic terms. The Bayesian information criterion was used to select the optimal number and form of trajectories. GBTM was performed using the traj package in Stata software (StataCorp, College Station, TX).

After unsupervised modeling of longitudinal trajectories, we used supervised multivariable logistic regression to examine the independent associations between trajectory group memberships and overall and high-risk revisits, adjusting for potential confounders. Variables associated with the study outcome in univariate analyses and selected variables based on a review of the medical literature were considered for inclusion in the multivariable model.

Multicollinearity was checked by computing variance inflation factors. All VIFs were less than 10, suggesting low collinearity. The discriminatory ability and the goodness-of-fit of the model were evaluated using the area under the receiver operating characteristic curve (AUROC) and the Hosmer-Lemeshow test, respectively. We tested for a priori two-way interaction by multiplying the two factors of interest and including an interaction term in the final multivariable model. To validate the identified trajectory groups, we employed bootstrapping 100 times to obtain bias-corrected confidence intervals for the models. All odds ratios (ORs) and beta coefficients are presented with 95% CIs. All analyses were performed using Stata 16.0 software. All P values are two-sided, with *P* < 0.05 indicating statistical significance.

## Results

From January 2016 to December 2019, there were 454,330 adult ED visits (Fig. 1). After excluding those who were not discharged and whose vital signs were measured less than three times in the ED, there were 39,138 potential index ED visits. There were 3,201 patients who returned to the ED within 72 h, 78 of whom were high-risk revisits. The overall revisit rate was 8.2%, while the high-risk revisit rate was 0.2%.

Table 1 shows the differences in patient characteristics between the revisit group and the non-revisit group during their index visit. The mean age of the patients in both groups was similar (approximately 57 years), while female patients were predominant in the no-revisit group (53% vs. 50%). There were no differences in seasonal, weekend, or diurnal patterns at the time of ED presentation. However, the distribution of the subdivisions differed, with more medical patients in the revisit group. The prevalence of major diseases was higher in the revisit group (17%) than in the non-revisit group (11%); however, the revisit group was less likely to arrive by ambulance than the non-revisit group. Chief complaints also varied significantly between the groups, with abdominal pain, fever, and dyspnea being more prevalent in the revisit group. Triage levels demonstrated a notable distinction, with a smaller percentage of sicker patients (triage levels 1 and 2, 23%) in the revisit group than in the no-revisit group (26%). The ED length of stay (LOS) was longer for the revisit group during their index visit, with a median of 12.0 h, compared to 7.0 h for the no-revisit group. There were no differences between the two groups regarding the age and gender of the treating physician or discharge timing.

**Table 1:** Baseline clinical characteristics of emergency department patients by revisit status

Variable	Revisit Group *N* = 3,201	Non-revisit Group *N* = 35,937	*P* value
Age, mean (SD), yr	57.8 (20.4)	57.5 (20.6)	0.412
Female sex, n (%)	1,609 (50.3)	19,049 (53.0)	0.003
Season, n (%)			0.778
Spring (Mar. – May)	778 (24.3)	8,714 (24.3)	
Summer (Jun. – Aug.)	844 (26.4)	9,401 (26.2)	
Fall (Sep. – Nov.)	872 (27.2)	9,608 (26.7)	
Winter (Dec. – Feb.)	707 (22.1)	8,214 (22.9)	
Weekend, n (%)	887 (27.7)	9,901 (27.6)	0.865
Presenting Time, n (%)			0.081
7:00 am to 2:59 pm	1,105 (34.5)	12,916 (35.9)	
3:00 pm to 10:59 pm	1,310 (40.9)	14,778 (41.1)	
11:00 pm to 6:59 am	786 (24.6)	8,243 (22.9)	
Subdivision, n (%)			< 0.001
Medicine	2,707 (84.6)	30,071 (83.7)	
Trauma	423 (13.2)	5,417 (15.1)	
Ob/Gyn	11 (0.3)	107 (0.3)	
Other	60 (1.9)	342 (1.0)	
Major disease, n (%)	549 (17.2)	3,979 (11.1)	< 0.001
Arrival by ambulance, n (%)	436 (13.6)	7,129 (19.8)	< 0.001
Chief complaint, n (%)			< 0.001
Abdominal pain	435 (13.6)	3,966 (11.0)	
Dizziness	159 (5.0)	2,784 (7.8)	
Chest pain	193 (6.0)	2,379 (6.6)	
Fever	163 (5.1)	1,309 (3.6)	
Dyspnea	126 (3.9)	986 (2.7)	
Other	2,127 (66.4)	24,511 (68.2)	
Triage level, n (%)			< 0.001
1	70 (2.2)	919 (2.6)	
2	655 (20.5)	8,463 (23.6)	
3	2,351 (73.5)	25,586 (71.2)	
4	117 (3.7)	887 (2.5)	
5	8 (0.3)	61 (0.2)	
ED length of stay, median (IQR), hr	12.0 (6.0–22.0)	7.0 (5.0–12.0)	< 0.001
Age of treating physician, mean (SD), year	36.8 (7.0)	37.0 (7.2)	0.069
Sex of treating physician, n (%)			0.377
Male	2,879 (90.0)	32,133 (89.5)	
Female	321 (10.0)	3,782 (10.5)	
Discharged on weekends, n (%)	905 (28.3)	10,194 (27.4)	0.910
Discharge Time, n (%)			0.661
7:00 am to 2:59 pm	1,307 (40.8)	14,455 (40.2)	
3:00 pm to 10:59 pm	1,140 (35.6)	13,085 (36.4)	
11:00 pm to 6:59 am	754 (23.6)	8,397 (23.4)	

Abbreviations: SD = Standard deviation; IQR = Interquartile range; ED = Emergency department

Table 2 shows the differences in patient characteristics between the high-risk revisit group and the other group (non-high-risk revisits and no revisits) upon discharge at the index visit. Patients in the high-risk revisit group had a significantly higher mean age (69 years) than those in the other group (58 years), but the gender distribution was similar between the two groups. There were no differences in seasonal, weekend, or diurnal patterns at the time of ED presentation. The high-risk revisit group exhibited a greater prevalence of major disease (28.2%) than the other group (11.5%). Subdivision distribution, arrival by ambulance, or chief complaint did not differ between the groups. Triage levels showed a significant difference between the groups, with the high-risk revisit group being triaged to higher levels (levels 1 + 2) during their index visits. No significant differences were observed between the two groups in ED LOS, age or gender of the treating physician, or discharge timing.

**Table 2:** Baseline clinical characteristics of emergency department patients by high-risk revisit status

Variable	High-risk Revisit *N* = 78	Other (Non-high-risk revisit and no revisit) *N* = 39,060	*P* value
Age, mean (SD), yr	68.6 (18.6)	57.5 (20.6)	< 0.001
Female sex, n (%)	41 (52.6)	20,617 (52.8)	0.969
Season, n (%)			0.621
Spring (Mar. – May)	22 (28.2)	9,470 (24.2)	
Summer (Jun. – Aug.)	22 (28.2)	10,223 (26.2)	
Fall (Sep. – Nov.)	16 (20.5)	10,464 (26.8)	
Winter (Dec. – Feb.)	18 (23.1)	8,903 (22.8)	
Presenting Time, n (%)			0.886
7:00 am to 2:59 pm	30 (38.5)	13,991 (35.8)	
3:00 pm to 10:59 pm	31 (39.7)	16,057 (41.1)	
11:00 pm to 6:59 am	17 (21.8)	9,012 (23.1)	
Presenting on weekends, n (%)	28 (35.9)	10,759 (27.5)	0.099
Subdivision, n (%)			0.605
Medicine	69 (44.5)	32,709 (83.7)	
Trauma	9 (11.5)	5,831 (14.9)	
Ob/Gyn	0 (0.0)	118 (0.3)	
Other	0 (0.0)	402 (1.0)	
Major disease, n (%)	22 (28.2)	4,506 (11.5)	< 0.001
Arrival by ambulance, n (%)	15 (19.2)	7,550 (19.3)	0.982
Most common chief complaint, n (%)			0.600
Abdominal pain	6 (7.9)	4,395 (11.3)	
Dizziness	4 (5.3)	2,939 (7.5)	
Chest pain	7 (9.2)	2,565 (6.6)	
Fever	1 (1.4)	1,471 (3.9)	
Dyspnea	2 (2.6)	1,110 (2.8)	
Other	56 (73.7)	26,582 (68.1)	
Triage level, n (%)			0.004
1	5 (6.4)	984 (2.5)	
2	29 (37.2)	9,089 (23.3)	
3	41 (52.6)	27,896 (71.5)	
4	3 (3.9)	1,001 (2.6)	
5	0 (0.0)	69 (0.2)	
ED length of stay, median (IQR), hr	7.0 (5.0–13.0)	7.0 (5.0–12.0)	0.961
Age of treating physician, mean (SD), year	37.8 (5.5)	37.0 (7.2)	0.323
Sex of treating physician, n (%)			0.662
Male	71 (91.0)	34,941 (89.5)	
Female	7 (9.0)	4,096 (10.5)	
Discharged on weekends, n (%)	27 (34.6)	11,072 (28.4)	0.220
Discharge Time, n (%)			0.137
7:00 am to 2:59 pm	23 (29.5)	15,739 (40.3)	
3:00 pm to 10:59 pm	32 (41.0)	14,193 (36.3)	
11:00 pm to 6:59 am	23 (29.5)	9,128 (23.4)	

Abbreviations: SD = Standard deviation; IQR = Interquartile range; ED = Emergency department

**Fig. 2:**
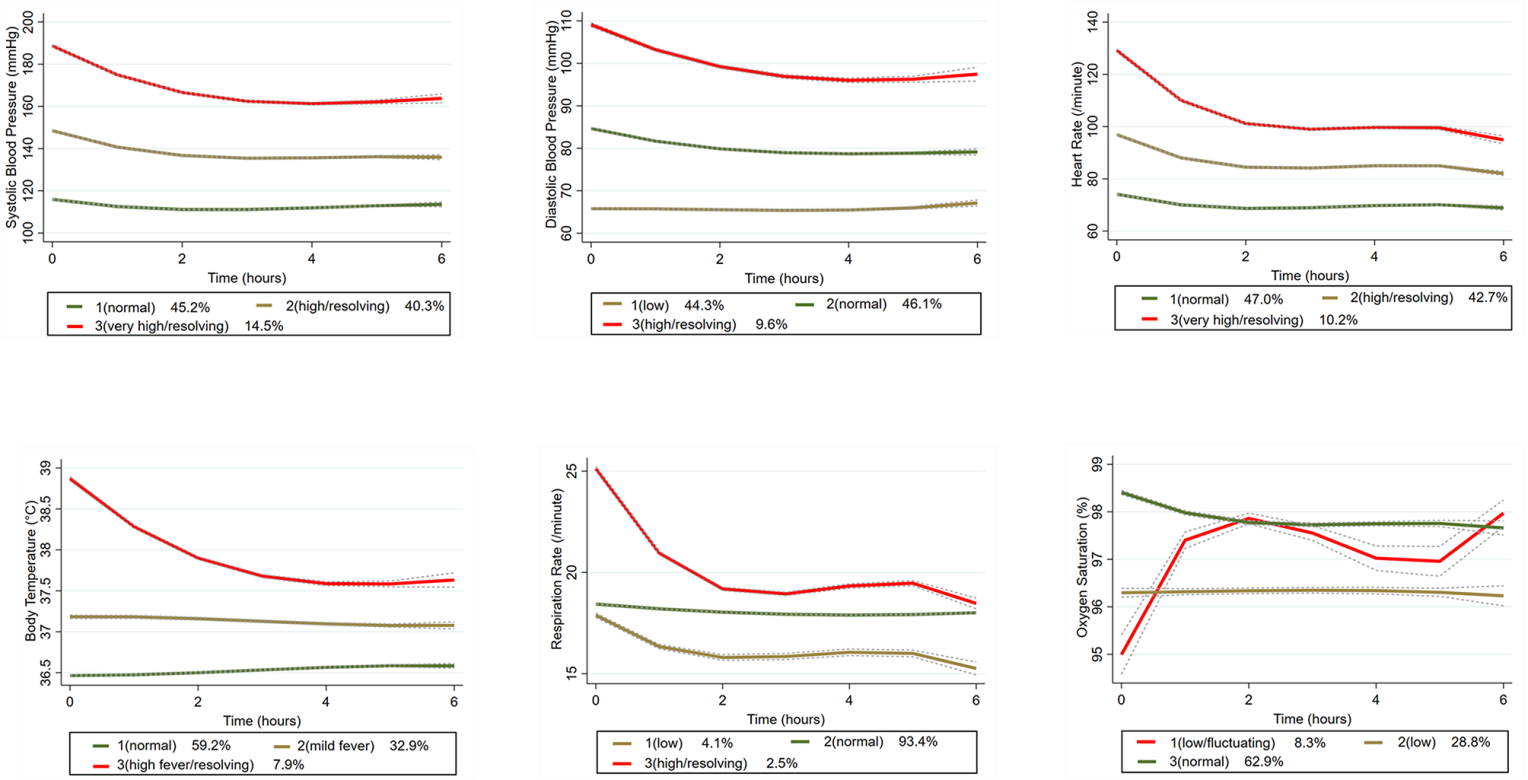
The vital sign trajectory groups identified by group-based trajectory modeling. The percentage in parenthesis denotes the proportion of patients in that trajectory group. The lines around the trajectory indicate the confidence intervals

Figure 2 depicts the trajectory groups in each vital sign category. Three trajectory groups per vital sign were identified via trajectory modeling. Based on the overall trajectory shapes (starting point and subsequent changes over time, if any), we used descriptive terms to name these trajectory groups. For example, in the SBP category, three distinct trajectory groups, namely, “normal” (45% of patients), “high/resolving” (40%), and “very high/resolving” (15%), were identified. In the DBP category, groups 1–3 corresponded to low, normal, and high/resolving DBP groups, respectively. In the HR category, groups 1–3 corresponded to normal, high/resolving, and very high/resolving HR groups. In the BT category, groups 1–3 corresponded to normal, mild, and high fever/resolving BT groups. In the RR category, groups 1–3 corresponded to low, normal, and high/resolving RR groups. In the SpO2 category, groups 1–3 corresponded to low/fluctuating, low, and normal oxygen saturation groups. The summary statistics (initial value, last value, and standard deviations) for each vital sign category are included in Online Supplementary Table S2.

Multivariable analysis (Table 3) revealed factors associated with overall revisits. Patients with a low DBP trajectory during the index stay were less likely to revisit the ED overall. In contrast, patients with a high fever/resolving BT trajectory were more likely to revisit the ED. Female patients exhibited a lower likelihood of revisits. The subdivision was also a significant factor, with patients in the “other” subgroup (e.g., psychiatry) having greater odds of revisiting than those in the medical subdivision. The presence of major diseases significantly increased the odds of revisits; in contrast, arrival by an ambulance was associated with decreased odds of revisits. Chief complaints of abdominal pain and dyspnea contribute to higher odds of revisits, while complaints of dizziness are associated with lower odds. Triage level 2 was associated with a lower possibility of revisits, while level 4 was associated with a higher likelihood of revisits. A longer ED LOS was correlated with higher overall revisit risk. The AUROC for the final model was 0.69, and the Hosmer-Lemeshow test was statistically significant (*P* < 0.01). The ROC curve was included in Online Supplementary Figure. We tested two clinically meaningful interactions (DBP by sex and BT by major disease), but neither of them was statistically significant. In other words, the association between DBP and overall revisits did not vary by sex. In addition, the association between BT and overall revisits did not vary by the presence of major disease.

**Table 3:** Multivariable model of trajectory groups associated with emergency department revisits (overall)

Variable	Adjusted Odds Ratio	95% Confidence Interval	*P* value
DBP trajectory group			
1 (low)	0.89	0.81–0.97	**0.009**
2 (reference)	1.00		
3 (high/resolving)	0.99	0.86–1.15	0.927
BT trajectory group			
1 (reference)	1.00		
2 (mild fever)	1.08	0.99–1.18	0.073
3 (high fever/resolving)	1.32	1.13–1.53	**< 0.001**
Female sex	0.92	0.85–0.99	**0.035**
Subdivision			
Medicine (reference)	1.00		
Trauma	1.05	0.94–1.19	0.388
Ob/Gyn	1.35	0.71–2.54	0.357
Other (e.g., psychiatry)	1.45	1.07–1.95	**0.015**
Major disease	1.59	1.43–1.76	**< 0.001**
Arrival by ambulance	0.59	0.52–0.66	**< 0.001**
Chief complaint			
Abdominal pain	1.27	1.13–1.43	**< 0.001**
Dizziness	0.74	0.62–0.88	**0.001**
Chest pain	1.10	0.93–1.30	0.273
Fever	1.11	0.92–1.35	0.270
Dyspnea	1.34	1.09–1.66	**0.006**
Other (reference)	1.00		
Triage level			
1	0.80	0.60–1.06	0.117
2	0.90	0.81–0.99	**0.035**
3 (reference)	1.00		
4	1.35	1.10–1.67	**0.004**
5	1.29	0.61–2.75	0.502
ED LOS	1.06	1.06–1.06	**< 0.001**

Significant odds ratios are highlighted in bold

Abbreviations: DBP = Diastolic blood pressure; BT = Body temperature; ED LOS = Emergency department length of stay

Model adjusted for age, sex, six vital sign trajectory groups, subdivision, major disease, arrival by ambulance, chief complaint, and triage levels, chief complaint, and emergency department length of stay

Statistically non-significant predictors are not shown

Table 4 summarizes findings from a multivariable analysis of factors associated with high-risk ED revisits. A low/fluctuating oxygen saturation trajectory (adjusted odds ratio [aOR], 2.40; 95% CI, 1.15–4.99) was associated with high-risk revisits. Older age (aOR, 1.27; 95% CI, 1.11–1.46) and having a major disease (aOR, 2.30; 95% CI, 1.38–3.84) were also associated with a high likelihood of high-risk revisits. The AUROC for the final model was 0.72, and the Hosmer-Lemeshow test was statistically significant (*P* = 0.02). The ROC curve was included in Online Supplementary Figure. We tested two clinically meaningful interactions (SpO_2_ by age and SpO_2_ by major disease), but neither of them was statistically significant. In other words, the association between SpO2 and high-risk revisits did not vary by age or the presence of major disease.

**Table 4:** Multivariable model of trajectory groups associated with emergency department revisits (high risk)

Variable	Adjusted Odds Ratio	95% Confidence Interval	*P* value
SpO_2_ trajectory group			
1 (low/fluctuating)	2.40	1.15–4.99	**0.020**
2 (low)	1.37	0.81–2.30	0.237
3 (reference)	1.00		
Age, per 10-year increase	1.27	1.11–1.46	**0.001**
Major disease	2.30	1.38–3.84	**0.001**

Significant odds ratios are highlighted in bold

Abbreviations: SpO_2_ = Oxygen saturation

Model adjusted for age, sex, major disease, and all six trajectory groups. Statistically non-significant predictors are not shown

## Discussion

This retrospective analysis of 454,330 ED visits from January 2016 to December 2019 highlights the patient characteristics and vital sign trajectories associated with overall and high-risk ED revisits. Understanding these factors may help emergency physicians improve patient care and safety, potentially intercepting inappropriate and unsafe ED discharge.

### Revisit rate

The overall revisit rate to the ED was 8.2%, with a high-risk revisit rate of 0.2%. This overall rate appears to be slightly greater than that in previous studies [2, 3, 9, 16–19], suggesting potential variations attributable to differences in health care accessibility and disease complexities across different studies. Specifically, Taiwan has universal health insurance coverage with relatively inexpensive ED care costs, which may contribute to the higher revisit rate [20]. In addition, the hospitals in the current study are located in urban areas, and patients in the catchment area were relatively closer to the hospitals, which may also increase the revisit rate [21]. In contrast, the high-risk revisit rate of 0.2% is comparable to that reported in previous studies, indicating the necessity of ED visits among these critical patients regardless of health care system design [12, 18, 22].

### Older age as a predictor of high-risk revisits; male sex as a predictor of overall revisits

Patients in the high-risk revisit group demonstrated an older mean age of 68.6 years. With each 10-year increase in age, there was also a corresponding increase in the odds of high-risk revisits in the multivariable analysis. Older patients may experience more rapid disease progression, present with atypical symptoms, or exhibit greater tolerance for perceived illness, making them more prone to high-risk revisits [18, 23–25]. Gender differences were also noted, with a higher proportion of males in the overall revisit group. The higher overall revisit rate among men aligns with previous research findings, possibly due to poorer medical compliance among male patients [26].

### Major disease as a predictor of overall and high-risk revisits

The prevalence of major diseases is a crucial factor, highlighting its important role in risk stratification in the ED. Those with major diseases demonstrated a significantly greater revisit rate, both in the overall revisit group and in the high-risk revisits group. This emphasizes the need for emergency physicians to be especially attentive to patients with major diseases when making discharge decisions and implement proactive measures to prevent potentially fatal outcomes [27, 28].

### Vital sign trajectories as predictors of overall and high-risk revisits

The analysis of vital sign trajectory groups revealed specific associations with overall and high-risk revisits. Patients exhibiting a high fever with a resolving trajectory were found to have increased odds of overall revisits, suggesting the potential utility of continuous vital sign monitoring for predicting future events. If possible, a 24-hour afebrile period may be a safer criterion before discharging these patients. Interestingly, a relatively low DBP may serve as a protective factor against overall revisits, suggesting that a low DBP may be a proxy for physical fitness [29]. A low/fluctuating SpO_2_ trajectory also proved to be a significant predictor of high-risk revisits. Thus, it is important for clinicians to vigilantly monitor changes in oxygen saturation during the patient’s stay in the ED, particularly for those deemed high-risk but about to be discharged. Alternatively, a snapshot of certain abnormal vital signs in the ED may hold a certain predictive value for ED revisits. For example, SBP < 120 mm Hg and HR > 90 beats/min in the ED have been reported as risk factors for high-risk returns [30, 31].

### Additional predictors of overall revisits

Additional predictors of overall revisits included subdivision, arrival by ambulance, and chief complaints. Patients registered in “other” departments (such as psychiatry) face challenges in obtaining specialized outpatient care, potentially contributing to subsequent return visits [25, 32, 33]. Patients with a major disease may have frequented ED for minor illnesses (leading to overall revisits), and this type of care did not necessitate ambulance transportation, as observed in this study. Chief complaints such as abdominal pain, fever, or dyspnea may present challenges due to the involvement of multiple organs/systems, complicating the identification of a specific cause at the index visit [22, 26, 28, 34, 35]. Physician characteristics, including age and gender, did not significantly differ between the groups, suggesting that revisit risk may be more influenced by patient-related factors than physician attributes. This finding was somewhat reassuring, as a recent study indicated that patients seen by older physicians in the ED had a higher post-ED mortality rate [36]. Other physician characteristics, such as years of experience, medical specialty, or treatment considerations, may also impact patient outcomes and revisit rates. Further research is needed to better understand how additional physician factors might affect ED revisit rates.

### Future directions

Future studies should focus on several key areas. First, the identified vital sign trajectories and their association with high-risk revisits require external validation. We are in the process of obtaining more recent data and data from other hospitals to validate our findings. Regarding the clinical utility of these trajectory findings, besides providing qualitative terms for the trajectories associated with overall or high-risk revisits, along with visual trajectory plots, we also provided the summary statistics for each trajectory as a reference. A more sophisticated approach would be a real-time prediction of the “closeness/fitness” of actual vital sign values during the ED stay to the at-risk trajectories identified in our study. This would require calculations of the posterior probability of group membership using all available data up to time t and updating it as new data comes in [37], and we are in the process of developing such an application. Second, as ED processes and patient care protocols may have evolved, especially due to technological advancements and external factors such as the COVID-19 pandemic, it is crucial to investigate whether our findings remain in the post-COVID era. Lastly, while our study focused on early high-risk revisits, future research should explore the long-term health outcomes and healthcare utilization of these patients, as early revisits may indicate unmet medical needs and require longer-term transitional care planning.

This study has some potential limitations. First, this was a single-center study at a tertiary medical center, and our findings may not be generalizable to hospitals in different settings. Second, our study population was restricted to those who underwent at least three vital sign measurements to ensure the stability of the statistical analysis. Therefore, patients with mild symptoms may be excluded due to a lower frequency of measurements, making the study results potentially not applicable to these patients. Nonetheless, we included an analysis of high-risk revisits among those who were discharged at the index visit (without the requirement of three vital sign measurements) for interested readers (Online Supplementary Table S3). Third, we did not control for treatment effects such as oxygen administration or antipyretics, which could have influenced the trajectories. However, this made our results potentially more representative of a real-world scenario. Fourth, we did not consider individual classifications of major diseases, socioeconomic status, patients’ proximity to the hospital, patient behavior (e.g., adherence to discharge instructions), or physician experience and specialty that may have influenced ED revisits. Finally, variations in EHR data may introduce misclassification bias (likely non-differential), biasing the results toward the null.

## Conclusions

In summary, our study provides ED clinicians with information related to overall revisits and possible strategies to prevent high-risk ED visits. The identified predictors of high-risk revisits included specific vital sign trajectories, older age, and major diseases. In the digital health era, the novel trajectories identified in this study can be programmed into a hospital analytic platform to identify high-risk ED patients who may need longer observation or additional support if discharged from the ED, thereby reducing detrimental and costly ED revisits.

## Electronic supplementary material

Below is the link to the electronic supplementary material.

Supplementary Material 1

## Data Availability

The datasets used and/or analyzed during the current study are available from the corresponding author on reasonable request.

## Abbreviations

ED: Emergency department
ICU: Intensive care unit
IHCA: In-hospital cardiac arrest
SBP: Systolic blood pressure
DBP: Diastolic blood pressure
HR: Heart rate
BT: Body temperature
RR: Respiratory rate
SpO_2_: Oxygen saturation
TTAS: Taiwan Triage and Acuity Scale
CI: Confidence interval
SD: Standard deviation
IQR: Interquartile range
GBTM: Group-based trajectory modeling

## References

[1] Duseja R, et al. Revisit rates and associated costs after an emergency department encounter: A multistate analysis. Ann Intern Med. 2015;162(11):750–6. 26030633 10.7326/M14-1616

[2] Lu TC, et al. Emergency department revisits: A nation-wide database analysis on the same and different hospital revisits. Eur J Emerg Med. 2020;27(2):114–20. 31815872 10.1097/MEJ.0000000000000650

[3] Rising KL, et al. Patient returns to the emergency department: The time-to-return curve. Acad Emerg Med. 2014;21(8):864–71. 25154879 10.1111/acem.12442

[4] Abualenain J, et al. The prevalence of quality issues and adverse outcomes among 72-hour return admissions in the emergency department. J Emerg Med. 2013;45(2):281–8. 23352864 10.1016/j.jemermed.2012.11.012

[5] Calder L, et al. Adverse events in patients with return emergency department visits. BMJ Qual Saf. 2015;24(2):142–8. 25540424 10.1136/bmjqs-2014-003194 PMC4316869

[6] Cheng J, et al. Emergency department return visits resulting in admission: Do they reflect quality of care? Am J Med Qual. 2016;31(6):541–51. 26160967 10.1177/1062860615594879

[7] Easter JS, Bachur R. Physicians’ assessment of pediatric returns to the emergency department. J Emerg Med. 2013;44(3):682–8. 22818645 10.1016/j.jemermed.2012.05.011

[8] Jiménez-Puente A, et al. Which unscheduled return visits indicate a quality-of-care issue? Emerg Med J. 2017;34(3):145–50. 27671021 10.1136/emermed-2015-205603

[9] Sabbatini AK, et al. In-Hospital outcomes and costs among patients hospitalized during a return visit to the emergency department. JAMA. 2016;315(7):663–71. 26881369 10.1001/jama.2016.0649 PMC8366576

[10] Tsai CL, et al. Inpatient outcomes following a return visit to the emergency department: A nationwide cohort study. West J Emerg Med. 2021;22(5):1124–30. 34546889 10.5811/westjem.2021.6.52212 PMC8463058

[11] Aaronson E, et al. Unscheduled return visits to the emergency department with ICU admission: A trigger tool for diagnostic error. Am J Emerg Med. 2020;38(8):1584–7. 31699427 10.1016/j.ajem.2019.158430

[12] Sung CW, et al. Factors associated with a high-risk return visit to the emergency department: A case-crossover study. Eur J Emerg Med. 2021;28(5):394–401. 34191766 10.1097/MEJ.0000000000000851

[13] Ng CJ, et al. Validation of the Taiwan triage and acuity scale: A new computerised five-level triage system. Emerg Med J. 2011;28(12):1026–31. 21076055 10.1136/emj.2010.094185

[14] Mori M, Krumholz HM, Allore HG. Using latent class analysis to identify hidden clinical phenotypes. JAMA. 2020;324(7):700–1. 32808993 10.1001/jama.2020.2278

[15] Jones B, Nagin D. A note on a stata plugin for estimating group-based trajectory models. Sociol Meth & Res. 2013;42:608–13.

[16] Hong WS, Haimovich AD, Taylor RA. Predicting 72-hour and 9-day return to the emergency department using machine learning. JAMIA Open. 2019;2(3):346–52. 31984367 10.1093/jamiaopen/ooz019 PMC6951979

[17] Kim DU, et al. Influence of overcrowding in the emergency department on return visit within 72 hours. J Clin Med. 2020;9(5). 10.3390/jcm9051406 PMC7290478 32397560

[18] Ling DA, et al. High-risk return visits to united States emergency departments, 2010–2018. West J Emerg Med. 2022;23(6):832–40. 36409935 10.5811/westjem.2022.7.57028 PMC9683777

[19] Michelson KA, et al. Timing and location of emergency department revisits. Pediatrics. 2018;141(5). 10.1542/peds.2017-4087 29650806

[20] Cheng TM. Reflections on the 20th anniversary of Taiwan’s single-payer national health insurance system. Health Aff (Millwood). 2015;34(3):502–10. 25732502 10.1377/hlthaff.2014.1332

[21] Naseer M, et al. Individual and contextual predictors of emergency department visits among community-living older adults: A register-based prospective cohort study. BMJ Open. 2022;12(2):e055484. 35140159 10.1136/bmjopen-2021-055484 PMC8830250

[22] Hiti EA, et al. Characteristics and determinants of high-risk unscheduled return visits to the emergency department. Emerg Med J. 2020;37(2):79–84. 31806725 10.1136/emermed-2018-208343 PMC7027026

[23] Hammouda N, et al. Geriatric emergency department revisits after discharge with potentially inappropriate medications: A retrospective cohort study. Am J Emerg Med. 2021;44:148–56. 33621716 10.1016/j.ajem.2021.02.004 PMC12324923

[24] Naseer M, et al. Factors associated with emergency department revisits among older adults in two Swedish regions: A prospective cohort study. Arch Gerontol Geriatr. 2020;86:103960. 31704624 10.1016/j.archger.2019.103960

[25] Sheikh S. Risk factors associated with emergency department recidivism in the older adult. West J Emerg Med. 2019;20(6):931–8. 31738721 10.5811/westjem.2019.7.43073 PMC6860386

[26] Tsai IT, et al. Characteristics and outcomes of patients with emergency department revisits within 72 hours and subsequent admission to the intensive care unit. Ci Ji Yi Xue Za Zhi. 2016;28(4):151–6. 10.1016/j.tcmj.2016.07.002 PMC5442903 28757746

[27] Guo DY, et al. The association between emergency department revisit and elderly patients. J Acute Med. 2020;10(1):20–6. 32995151 10.6705/j.jacme.202003_10(1).0003 PMC7517912

[28] Hayward J, et al. Predictors of admission in adult unscheduled return visits to the emergency department. West J Emerg Med. 2018;19(6):912–8. 30429921 10.5811/westjem.2018.8.38225 PMC6225947

[29] Jeoung B. The relationship between blood pressure and functional fitness of older adults in Korea. J Exerc Rehabil. 2024;20(1):11–6. 38433856 10.12965/jer.2346596.298 PMC10902697

[30] Gabayan GZ, et al. Emergency department vital signs and outcomes after discharge. Acad Emerg Med. 2017;24(7):846–54. 28375565 10.1111/acem.13194 PMC5935002

[31] Gabayan GZ, et al. Poor outcomes after emergency department discharge of the elderly: A case-control study. Ann Emerg Med. 2016;68(1):43–e512. 26947799 10.1016/j.annemergmed.2016.01.007 PMC5310269

[32] Lyons TW, et al. Patients visiting multiple emergency departments: Patterns, costs, and risk factors. Acad Emerg Med. 2017;24(11):1349–57. 28861915 10.1111/acem.13304 PMC5681430

[33] Venkatesh AK, et al. Variation in US hospital emergency department admission rates by clinical condition. Med Care. 2015;53(3):237–44. 25397965 10.1097/MLR.0000000000000261 PMC4858175

[34] Kacprzyk A, et al. Analysis of readmissions to the emergency department among patients presenting with abdominal pain. BMC Emerg Med. 2020;20(1):37. 32398140 10.1186/s12873-020-00334-x PMC7216723

[35] Montoy JCC, et al. Predicting emergency department bouncebacks: A retrospective cohort analysis. West J Emerg Med. 2019;20(6):865–74. 31738713 10.5811/westjem.2019.8.43221 PMC6860392

[36] Miyawaki A, et al. Association between emergency physician’s age and mortality of medicare patients aged 65 to 89 years after emergency department visit. Ann Emerg Med. 2023;82(3):301–12. 36964007 10.1016/j.annemergmed.2023.02.010

[37] Nagin DS, Jones BL, Elmer J. Recent advances in group-based trajectory modeling for clinical research. Annu Rev Clin Psychol. 2024;20(1):285–305. 38382118 10.1146/annurev-clinpsy-081122-012416

