# Supplementary material for: Factors associated with overall and high-risk return visits to the emergency department: a vital sign trajectory approach
Source: BMC Emerg Med. 2025 Apr 12;25:57. doi: 10.1186/s12873-025-01211-1 (PMC11993975; doi:10.1186/s12873-025-01211-1)

Online Supplementary Table S1. A List of major illnesses defined by the National Health Insurance in Taiwan and the percentage of each category.

1. Cancer
2. Hereditary deficiencies of clotting factors
3. Severe hemolytic or aplastic anemia
4. End-stage renal disease on dialysis
5. Systemic autoimmune diseases
6. Chronic psychiatric disorders or dementia
7. Congenital metabolic anomalies
8. Major organ congenital anomalies
9. Massive burn
10. Organ transplantation
11. Cerebral palsy
12. Major trauma rated 16 or above on the severity scale
13. Long-term mechanical ventilation dependency
14. Severe malnutrition on long-term parenteral nutrition
15. Decompression sickness
16. Myasthenia gravis
17. Congenital immunodeficiencies
18. Spinal cord injuries
19. Occupational lung disease
20. Cerebrovascular disease (acute stage)
21. Multiple sclerosis
22. Congenital muscular dystrophy
23. Congenital anomalies of skin
24. Leprosy (Hansen's disease)
25. Liver cirrhosis with complication
26. Premature infants with complications
27. Toxic effect of arsenic and its compounds (black foot disease)
28. Motor neuron disease
29. Creutzfeldt-Jakob disease
30. Rare disease

**Disease, n (%)**	**N = 990,896 (all major illness cardholders in Taiwan) in 2024**
Cancer	463,269 (46.8)
Hereditary deficiencies of clotting factors	1,856 (0.4)
Severe hemolytic or aplastic anemia	1,475 (0.2)
End-stage renal disease on dialysis	89,721 (9.1)
Systemic autoimmune diseases	139,826 (14.1)
Chronic psychiatric disorders or	189,102 (19.1)

dementia	
Congenital metabolic anomalies	18,388 (1.9)
Major organ congenital anomalies	37,868 (3.8)
Massive burn	292 (0.0)
Organ transplantation	17,465 (1.8)
Cerebral palsy	13,032 (1.3)
Major trauma rated 16 or above on the severity scale	12,485 (1.3)
Long-term mechanical ventilation dependency	11,196 (1.1)
Severe malnutrition on long-term parenteral nutrition	75 (0.0)
Decompression sickness	10 (0.0)
Myasthenia gravis	6,132 (0.6)
Congenital immunodeficiencies	218 (0.0)
Spinal cord injuries	7,459 (0.8)
Occupational lung disease	1,404 (0.1)
Multiple sclerosis	62 (0.0)
Congenital muscular dystrophy	367 (0.0)
Congenital anomalies of skin	131 (0.0)
Leprosy (Hansen's disease)	124 (0.0)
Liver cirrhosis with complication	3,602 (0.4)
Premature infants with complications	8 (0.0)
Toxic effect of arsenic and its compounds (black foot disease)	53 (0.0)
Motor neuron disease	96 (0.0)
Creutzfeldt-Jakob disease	20 (0.0)
Rare disease	15,171 (1.5)

Online Supplementary Table S2. The summary statistics (initial value, last value, and standard deviations) for each vital sign category.

	Mean of the initial value	SD of the initial value	Mean of the last value	SD of the last value
SBP Group (mmHg)				
1 Normal	115.4	17.0	113.0	13.9
2 High/resolving	149.3	17.8	137.2	17.2
3 Very high/resolving	189.7	21.8	165.6	22.9
DBP Group (mmHg)				
1 Low	65.1	11.0	66.4	9.5
2 Normal	85.1	10.8	78.9	10.4
3 High/resolving	110.0	16.0	98.1	15.0
HR Group (beats per min)				
1 Normal	73.8	11.0	69.6	9.7
2 High/resolving	97.8	12.1	84.4	11.5
3 Very high/resolving	130.5	20.5	98.3	16.6
BT Group (°C)				
1 Normal	36.5	0.5	36.5	0.4
2 Mild fever	37.3	0.5	37.1	0.5
3 High fever/resolving	38.9	0.7	37.6	0.8
RR Group (breaths per min)				
1 Low	18.5	1.4	17.7	1.6
2 Normal	20.8	3.3	20.7	2.6
3 High/resolving	25.6	2.9	18.5	1.6

SpO_2 Group (%)				
1 Low/fluctuating	93.2	7.2	98.2	3.3
2 Low	95.8	1.6	96.1	1.5
3 Normal	98.4	1.1	97.8	1.3

Abbreviations: SD = standard deviation; SBP = systolic blood pressure; DBP = diastolic blood pressure HR = heart rate; BT = body temperature; RR = respiratory rate; SpO_2 = oxygen saturation.

Online Supplementary Table S3. Baseline clinical characteristics of emergency department patients who are discharged at the index visit by high-risk revisit status.

**Variable**	**Revisit=344**	**Non-high-risk revisit=403,489**	**P value**
Age, mean (SD), yr	68.4 (17.1)	49.0 (20.3)	<0.001
Female sex, n (%)	168 (48.8)	220,813 (54.73)	0.028
Season, n (%)			0.615
Spring (Mar. – May)	83 (25.0)	101,022 (25.0)	
Summer (Jun. – Aug.)	94 (27.3)	101,620 (25.2)	
Fall (Sep. – Nov.)	73 (21.2)	95,480 (23.7))	
Winter (Dec. – Feb.)	94 (27.3)	105,367 (26.1)	
Presenting Time, n (%)			0.073
7:00 am to 2:59 pm	143 (41.6)	144,277 (35.8)	
3:00 pm to 10:59 pm	136 (39.5)	171,605 (42.5)	
11:00 pm to 6:59 am	65 (18.9)	87,607 (21.7)	
Presenting on weekends, n (%)	96 (27.9)	112,933 (28.0)	0.973
Subdivision, n (%)			<0.001
Medicine	251 (73.0)	251,849 (62.4)	
Trauma	92 (26.7)	137,087 (34.0)	
Ob/Gyn	0 (0)	7,341 (1.8)	
Other	1 (0.3)	7,212 (1.8)	
Major disease, n (%)	76 (22.1)	29,717 (7.4)	<0.001
Arrival by ambulance, n (%)	58 (16.9)	39,570 (9.8)	<0.001
Most common chief complaint, n (%)			0.002
Abdominal pain	34 (9.1)	39,257 (9.7)	
Dizziness	11 (3.0)	17,691 (4.4)	
Chest pain	21 (5.6)	12,212 (3.0)	
Fever	14 (3.8)	15,413 (3.8)	
Dyspnea	13 (3.5)	6,250 (1.6)	
Other	280 (75.1)	312,637 (77.5)	
Triage level, n (%)			<0.001
1	13 (3.8)	5,018 (1.2)	
2	91 (26.5)	44,560 (11.0)	

3	224 (65.1)	306,867 (76.1)	
4	13 (3.8)	43,091 (10.7)	
5	3 (0.9)	3,953 (1.0)	
Vital sign at triage			
Systolic blood pressure, mean (SD), mmHg	141.1 (30.6)	134.7 (26.5)	<0.001
Diastolic blood pressure, mean (SD), mmHg	78.0 (38.1)	78.9 (30.2)	0.651
Heart rate, mean (SD), beats per min	91.3 (21.6)	88.2 (29.1)	0.009
Body temperature, mean (SD), °C	37.2 (3.8)	37.6 (45.4)	0.110
Respiratory rate, mean (SD), breaths per min	20.3 (15.2)	18.6 (6.4)	0.039
Oxygen saturation, median (IQR), %	97.0 (95.0-98.0)	98.0 (97.0-99.0)	0.036
ED length of stay, median (IQR), hr	3.0 (2.0-6.0)	1.0 (1.0-3.0)	<0.001
Age of treating physician, mean (SD), year	37.4 (6.1)	36.2 (7.3)	0.001
Sex of treating physician, n (%)			0.158
Male	305 (88.7)	347,080 (86.0)	
Female	39 (11.3)	56,409 (14.0)	
Discharged on weekends	97 (28.2)	113,008 (28.1)	0.938
Discharge Time, n (%)			0.045
7:00 am to 2:59 pm	119 (34.6)	130,992 (32.5)	
3:00 pm to 10:59 pm	156 (45.4)	167,920 (41.6)	
11:00 pm to 6:59 am	69 (20.1)	104,577 (25.9)	

Abbreviations: SD = standard deviation; IQR = interquartile range; ED = emergency department;

Online Supplementary Figure. The receiver operating characteristic curves for the overall (left panel) and high-risk (right panel) revisit models.

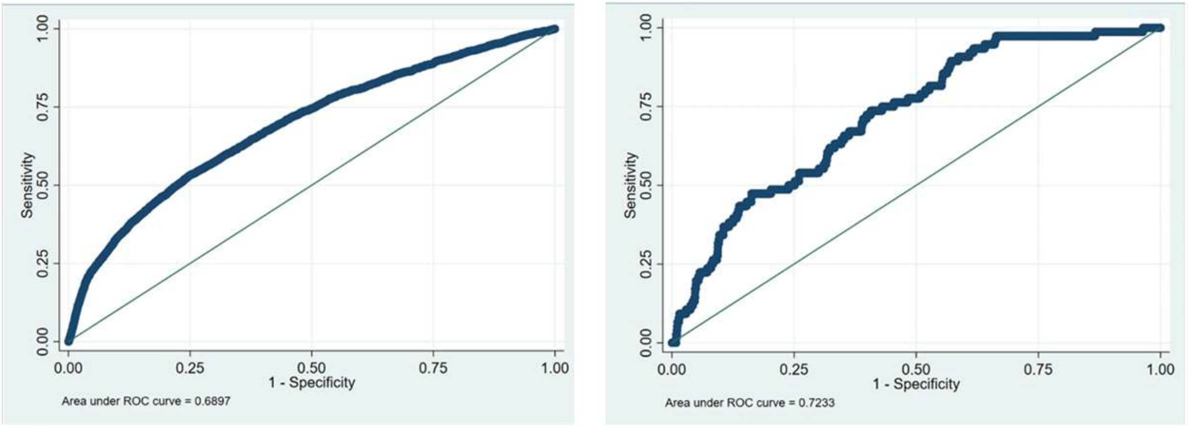

Supplement: Supplementary file 1 — Supplementary Material 1 [file 12873_2025_1211_MOESM1_ESM.pdf]
